# VE-821, an ATR inhibitor, causes radiosensitization in human tumor cells irradiated with high LET radiation

**DOI:** 10.1186/s13014-015-0464-y

**Published:** 2015-08-19

**Authors:** Hiroshi Fujisawa, Nakako Izumi Nakajima, Shigeaki Sunada, Younghyun Lee, Hirokazu Hirakawa, Hirohiko Yajima, Akira Fujimori, Mitsuru Uesaka, Ryuichi Okayasu

**Affiliations:** Department of Bioengineering, School of Engineering, The University of Tokyo, 7-3-1 Hongo, Bunkyo, Tokyo 113-8656 Japan; Research Center for Charged Particle Therapy/International Open Laboratory, National Institute of Radiological Sciences, 4-9-1 Anagawa, Inage, Chiba 263-8555 Japan; Department of Nuclear Engineering and Management, School of Engineering, The University of Tokyo, 7-3-1 Hongo, Bunkyo, Tokyo 113-8656 Japan; Research Center for Radiation Protection, National Institute of Radiological Sciences, 4-9-1 Anagawa, Inage, Chiba 263-8555 Japan

**Keywords:** ATR inhibition, Carbon ions, Cell cycle checkpoint, High LET radiation, VE-821

## Abstract

**Background:**

High linear energy transfer (LET) radiation such as carbon ion particles is successfully used for treatment of solid tumors. The reason why high LET radiation accomplishes greater tumor-killing than X-rays is still not completely understood. One factor would be the clustered or complex-type DNA damages. We previously reported that complex DNA double-strand breaks produced by high LET radiation enhanced DNA end resection, and this could lead to higher kinase activity of ATR protein recruited to RPA-coated single-stranded DNA. Although the effect of ATR inhibition on cells exposed to low LET gamma-rays has recently been reported, little is known regarding the effect of ATR inhibitor on cells treated with high LET radiation. The purpose of this study is to investigate the effects of the ATR inhibitor VE-821 in human tumor and normal cells irradiated with high LET carbon ions.

**Findings:**

HeLa, U2OS, and 1BR-hTERT (normal) cells were pre-treated with 1 μM VE-821 for 1 hour and irradiated with either high LET carbon ions or X-rays. Cell survival, cell cycle distribution, cell growth, and micronuclei formation were evaluated. VE-821 caused abrogation of G2/M checkpoint and forced irradiated cells to divide into daughter cells. We also found that carbon ions caused a higher number of multiple micronuclei than X-rays, leading to decreased cell survival in tumor cells when treated with VE-821, while the survival of irradiated normal cells were not significantly affected by this inhibitor.

**Conclusions:**

ATR inhibitor would be an effective tumor radiosensitizer with carbon ion irradiation.

**Electronic supplementary material:**

The online version of this article (doi:10.1186/s13014-015-0464-y) contains supplementary material, which is available to authorized users.

## Findings

### Introduction

More recently the use of charged ion particles for cancer therapy has been drawing attention. Especially, heavy charged particles such as carbon ions are successfully used for treatment of solid tumors [1, 2]. However, the reason why heavy ion particles have more tumor-killing effects than X-rays is not completely understood. DNA damage produced by high linear energy transfer (LET) radiation is considered to be qualitatively different from that produced by low LET radiation such as X-rays or protons. High LET radiation produces complex clustered damage, and its complexity of DNA damage may affect cell cycle progression, DNA repair pathways, and cell death [3].

We previously reported that the complex DNA double-strand breaks (DSBs) enhance DNA end resection during the repair process [4]. Other researchers showed that DNA end complexity determines the speed of DSB repair and the level of DSB end resection in G2 phase [5], and that cells irradiated with high LET Fe ions exhibited much greater G2/M accumulation than those with gamma-rays [6]. These results indicated that cells irradiated with high LET radiation show higher kinase activity of ataxia telangiectasia and Rad3-related (ATR) protein, because ATR is recruited to replication protein A (RPA)-coated single-stranded DNA (ssDNA) [7].

ATR is a DNA damage response kinase that is activated by DNA damage or replication stress to regulate the genomic integrity [8]. One of the ATR functions includes a cell cycle checkpoint after DNA DSBs. After DNA ends are processed by exonuclease, ATR is recruited to ssDNA and phosphorylates chk1, leading to cell cycle arrest in G2/M.

A recent report showed that VE-821, a novel ATR inhibitor, increased sensitivity to low LET radiation in pancreatic cancer cells [9]. This chemical is also known to inhibit chk1 phosphorylation [10, 11]. Although one report with another ATR inhibitor using normal cells irradiated with high LET radiation was recently published [12], no information with VE-821 using carbon ion irradiated tumor cells is available. Thus, we investigated the effect of VE-821 in tumor cells irradiated with carbon ions, and found that this drug is an effective radiosenstizer when combined with high LET radiation.

## Materials and methods

### Cell culture, IR irradiations, and drug treatments

HeLa, human cervical cancer cells, and U2OS, human osteosarcoma cells, were grown in MEM Eagle supplemented with 10 % fetal bovine serum plus antibiotics. HeLa cells were obtained from Cell Resource Center for Biomedical Research at Tohoku University, Sendai, Japan, and U2OS cells were obtained from ATCC (cell line #: HTB-96), USA. 1BR-hTERT, normal human fibroblasts kindly supplied by Dr. Jeggo, Sussex University, UK, were grown in DMEM supplemented with 15 % fetal bovine serum and antibiotics. All cells were maintained at 37 °C in a humidified CO_2_ incubator. 290 MeV/u carbon ions (LET of 70 keV/μm) and X-rays by TITAN-320 (200 kV, 20 mA, Shimadzu) were used for irradiation. Exponentially growing cells were pre-incubated with 1 μM ATR inhibitor VE-821 (Axon Medchem) or with DMSO for 1 hour before irradiation.

### Colony formation assay

At 8 or 24 hours after irradiation, cells were trypsinized, counted and plated onto cell culture dishes. After 12 days, cells were fixed in 100 % ethanol and stained with 0.1 % crystal violet. Colonies containing more than 50 cells were counted as surviving cells.

### Cell cycle distribution and cell growth analysis

Cells were irradiated with 3 Gy of carbon ions or 6 Gy of X-rays and harvested at 12 and 24 hours after irradiation. They were fixed with 70 % cold ethanol and stained with propidium iodide (PI) (50 μg/ml in PBS containing 200 μg/ml RNase) for 30 minutes. Then, they were analyzed on a FACSCalibur (Becton Dickinson) flow cytometer. For cell growth analysis, the number of cells was counted by Coulter Counter (Beckman Coulter) immediately and 1 day after irradiation.

### Micronucleus assay

Immediately after irradiation of U2OS cells with 1 Gy of carbon ions or 1 or 2 Gy of X-rays, and 1BR-hTERT cells with 2 Gy of X-rays, 2 μg/ml of cytochalasin-B (Cyt-B) was added and cells were incubated for 24 hours. They were then fixed in cold methanol for 20 minutes, permeabilized in 0.5 % Triton X-100 for 10 minutes on ice, and blocked in 3 % bovine serum albumin for 20 minutes at room temperature. They were incubated with anti-β-actin conjugated with Alexa Fluor 488 antibody (Cell Signaling) for 1 hour, and mounted in ProLong Gold Antifade with DAPI (Molecular Probe).

## Results

### The ATR inhibitor VE-821 radiosensitized human tumor cells irradiated with high LET carbon ions

To assess ATR inhibition on radiation sensitivity, cellular survivals were acquired using a colony formation assay. Relative biological effectiveness (RBE) based on D_10_ values, at a dose resulting in one decade of cell killing, was calculated as X-ray dose leading to 0.1 survival divided by carbon dose leading to 0.1 survival (Table 1).

**Table 1:** D_10_ with DMSO and RBE

	8 h			24 h		
	D_10_ DMSO (Gy)		RBE	D_10_ DMSO (Gy)		RBE
	X-ray	Carbon		X-ray	Carbon	
HeLa	5.47	2.67	2.05	6.66	2.62	2.54
U20S	3.81	1.64	2.31	4.01	1.64	2.45
1BR-hTERT	3.67	1.90	1.93	3.31	1.79	1.85

**Fig. 1:**
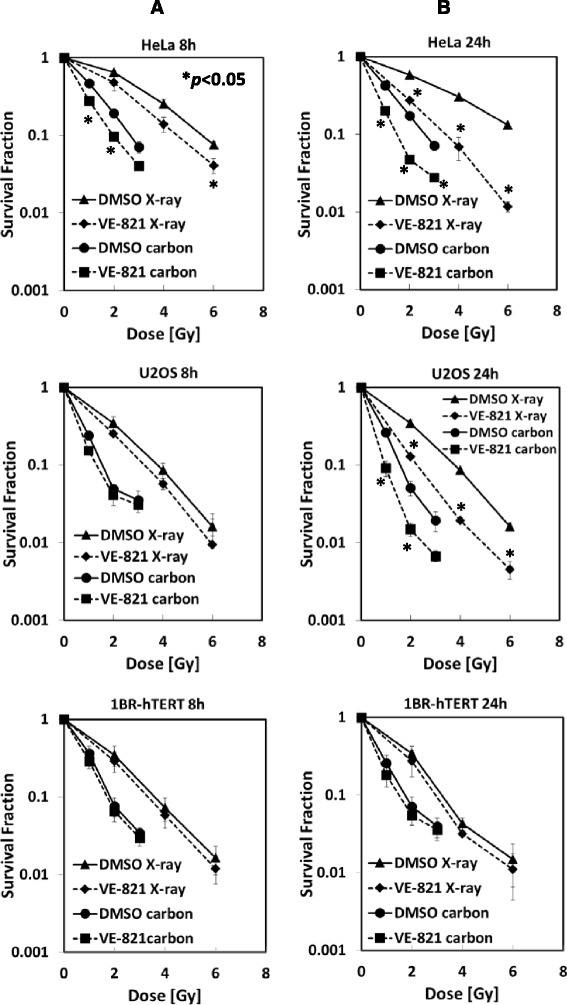
The ATR inhibitor VE-821 radiosensitized human tumor cells irradiated with high LET carbon ions. HeLa, U2OS, and 1BR-hTERT cells were pre-treated with 1 μM VE-821 or DMSO for 1 hour before irradiation, and were irradiated with 1, 2, 3 Gy of carbon ions or 2, 4, 6 Gy of X-rays. At 8 hours (**a**) or 24 hours (**b**) after irradiation, cells were plated on cell culture dishes to form colonies. Colonies containing more than 50 cells were counted as surviving cells. Error bars represent standard error of the mean (SEM) of at least three independent experiments. Curves with *show statistically significant data (p <0.05) when compared with control curves with DMSO only

With 8-hour treatment of VE-821, HeLa cells were clearly radiosensitized both with carbon ion and X-ray irradiation, while normal 1BR-hTERT cells were not affected (Fig. 1a). U2OS tumor cells were slightly affected by VE-821 with 8-hour treatment. Sensitizing enhancement ratios (SER) on the D_10_ value in carbon ion irradiation are shown in Table 2. With 24-hour treatment of VE-821, HeLa and U2OS cells were significantly radiosensitized by both kinds of radiation, while little was observed in irradiated 1BR-hTERT cells by the inhibitor (Fig. 1b).

**Table 2:** D_10_ withVE-821 and SER

	8 h				24 h			
	D_10_ VE-821 (Gy)		SER		D_10_ VE-821 (Gy)		SER	
	X-ray	Carbon	X-ray	Carbon	X-ray	Carbon	X-ray	Carbon
HeLa	4.52	1.96	1.21	1.36	3.45	1.50	1.93	1.74
U20S	3.25	1.28	1.17	1.29	2.25	0.95	1.78	1.72
1BR-hTERT	3.38	1.78	1.08	1.07	3.05	1.46	1.09	1.23

### VE-821 abrogated carbon ion-induced G2/M cell cycle arrest

**Fig. 2:**
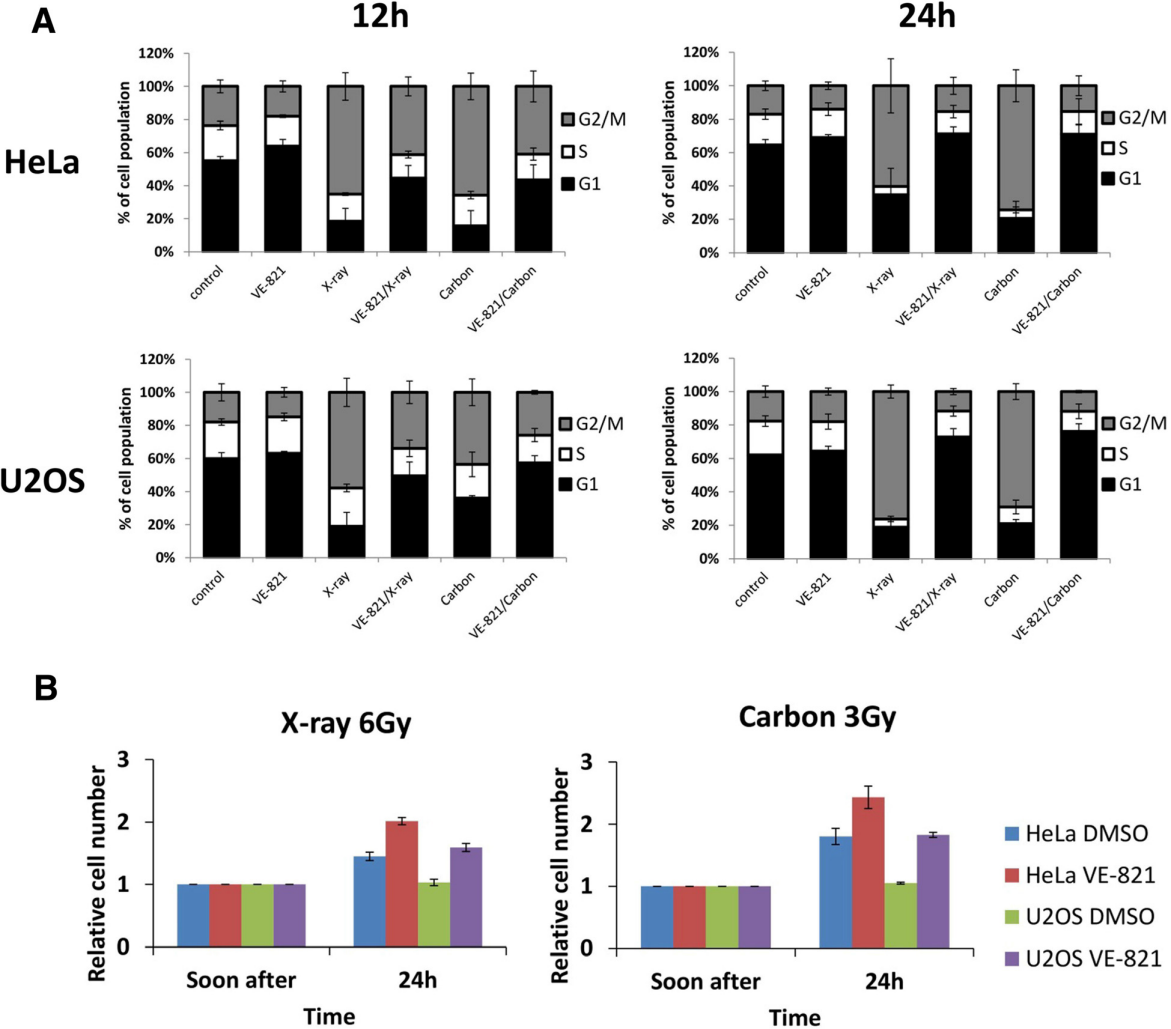
VE-821 abrogated carbon ion-induced G2/M cell cycle arrest in tumor cells. HeLa and U2OS cells were pre-treated with 1 μM VE-821 or DMSO for 1 hour before irradiation, and were irradiated with 3 Gy of carbon ions or 6 Gy of X-rays. **a** Cells were harvested at 12 and 24 hours after irradiation and their cell cycle distributions were analyzed by flow cytometry. **b** The number of cells was counted immediately after and 1 day after irradiation. The relative cell number was calculated as follows: cell number after 1 day was divided by cell number immediately after irradiation. Error bars represent SEM of at least three independent experiments

To investigate the effect of VE-821 on the cell cycle checkpoint after irradiation, we examined the cell cycle distribution by flow cytometer. Following carbon ion irradiation, all three cell lines showed significant G2/M checkpoint arrest at 12 hours after irradiation. However, the combination with VE-821 decreased the percentage of G2/M phase, indicating abrogation of G2/M cell cycle arrest. At 24 hours after irradiation, G2/M arrest continued in cells without the drug treatment, while cells with the combination treatment showed high percentages of G1 cells, the level of which was similar to, or even higher than non-irradiated control (Fig. 2a). Irradiated 1BR-hTERT cells showed a decrease in the percentage of S phase and an abrogation of G2/M cell cycle arrest (Additional file 1: Figure S1). There were no significant differences in VE-821 effect between the results with 3 Gy carbon ions and with 6 Gy X-rays, reflecting an RBE value of about 2 for carbon ions. Cellular growth data seem to indicate that VE-821 forced cells to divide into daughter cells (Fig. 2b).

### Carbon ions with VE-821 caused multiple micronuclei formation

**Fig. 3:**
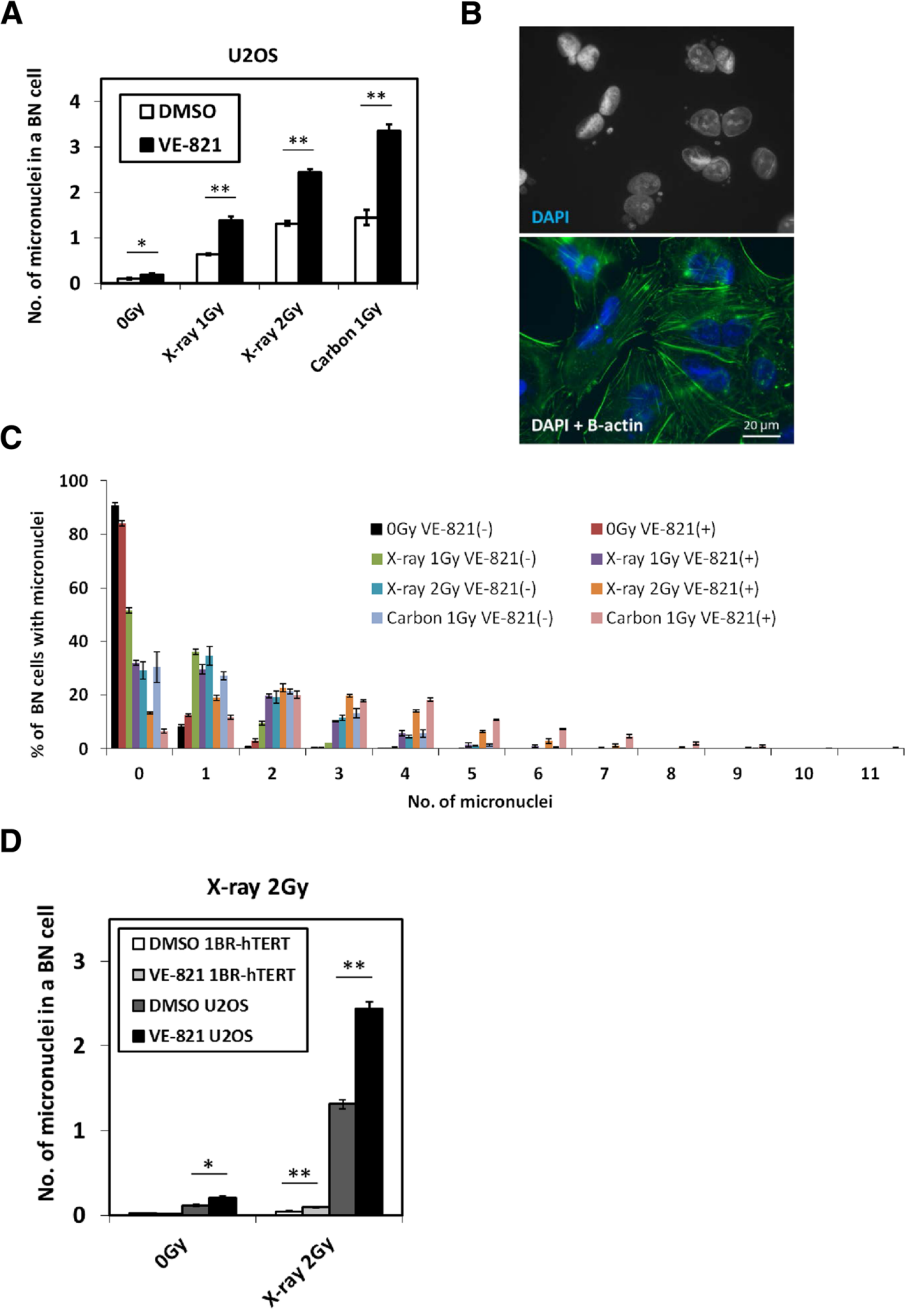
Carbon ions with VE-821 caused multiple micronuclei formation in tumor cells. **a** U2OS cells were pre-treated with 1 μM VE-821 or DMSO for 1 hour before irradiation, and were irradiated with 1 Gy of carbon ions, or 1 or 2 Gy of X-rays. They were treated with cytochalasin-B (2 μg/ml) immediately after irradiation and incubated for 24 hours. Nucleus and β-actin were stained with DAPI and anti-β-actin antibody. 500 binucleated (BN) cells were scored each time, and this was repeated three times (1500 BN cells were scored in total). Asterisk(s) in this figure means statistically significant (*: p < 0.05, **: p < 0.01) when the data under the bar were compared. **b** Microscopic images of U2OS cells irradiated with 1 Gy of carbon ions and VE-821. Cellular nuclei were stained with DAPI, and β-actin was stained with anti-β-actin conjugated with Alexa Fluor 488 antibody to detect the cellular surfaces. **c** Percentages of BN cells with micronuclei were shown as a function of micronucleus number. **d** The number of micronuclei in a BN cell was counted in U2OS and 1BR-hTERT cells with the combination of 2 Gy of X-ray irradiation and DMSO or VE-821 treatment. Error bars represent SEM of at least three independent experiments. Asterisks indicate the same meaning as in Fig. 3a

A micronucleus assay of U2OS cells was conducted to investigate genomic toxicity when treated with VE-821. In carbon ion-irradiated cells with VE-821, the average number of micronuclei per binucleated (BN) cell was 3.36 which is about 2.32 times higher than that treated with DMSO control. In X-ray-irradiated cells with VE-821, the average number was 1.38 and 2.44 at a dose of 1 and 2 Gy, respectively, 2.39 times and 1.86 times higher, respectively, than that with DMSO control (Fig. 3a, 3b). From the results of the percentage of BN cells with micronuclei, a substantial number of BN cells with multiple micronuclei were observed in the combination of VE-821 and irradiation for U2OS tumor cells (Fig. 3c). We also measured micronuclei formation using normal 1BR-hTERT cells. As indicated in Fig. 3d, the micronuclei formation is significantly lower in irradiated normal cells with and without VE-821 treatment when compared with U2OS tumor cells.

## Discussion

In this report we have shown for the first time that pre-treatment of the ATR inhibitor VE-821 clearly enhanced the effect of carbon ion irradiation in human tumor cells. In contrast, little radiosensitization was observed in normal human cells. Xue et al. [12] showed some radio-sensitization in different normal human cell line (GM0639) with carbon ions using another ATR inhibitor NU6027, but the concentration of the drug is 10 μM which is ten times higher than this study with VE-821. It appears that much lower concentration of VE-821 provides desirable differential effect between normal and tumor cells.

In cell cycle and growth analysis, we demonstrated that VE-821 abrogated G2/M cell cycle arrest, forcing cells to enter mitotic stage. Differences among the three cell lines were observed in the percentage of G1 phase at 12 hours after irradiation. The percentage of G1 HeLa cells after irradiation was less than those of U2OS and 1BR-hTERT. This result may be related to the p53 protein status. U2OS and 1BR-hTERT cells, expressing normal wild-type p53 protein, manifest both G1/S and G2/M checkpoint arrest, while HeLa has no or little G1/S checkpoint mechanism because HeLa cells express p53, but it is degraded by E6 proteins [13]. The cell growth data at 1 day after irradiation also showed little cell cycle arrest when treated with VE-821.

The effects of VE-821 on low LET radiation [11] as well as VE-821 on Gemcitabine treatment have been reported [9]. Effectiveness of this drug under hypoxic condition was also shown using 3-dimensional spheroids [14]. In our study SER depended on the treatment time of ATR inhibitor after irradiation. Much higher SER values were obtained at 24 hour than 8 hour post-irradiation; this trend is more pronounced in tumor cells than normal cells. By micronucleus analysis, similar numbers of micronuclei were observed for 1 Gy of carbon-irradiated cells and 2 Gy X-ray-irradiated cells. This might reflect an RBE value of carbon ions of around 2. Judging from the comparison of micronuclei formation between tumor and normal cells in Fig. 3d, it seems that tumor cells are much more genomically unstable, leading to higher number of chromosome fragmentations than normal cells, especially when irradiated cells are forced to advance from G2 to mitotic stage by VE-821 treatment. This manifests in the differences in degree of radio-sensitization between tumor and normal cells in Fig. 1, where only tumor cells were significantly radio-sensitized by VE-821. When ATR activity was inhibited, high LET radiation caused more multiple micronuclei in the BN tumor cells. Our studies indicated that the combination of ATR inhibitor and high LET carbon irradiation provided very effective tumor control. Thus, ATR inhibition would be a good alternative for the combined treatment with low and high LET radiation.

## Additional file

Additional file 1: Figure S1. VE-821 abrogated carbon ion-induced G2/M cell cycle arrest in 1BR-hTERT cells. 1BR-hTERT cells were pre-treated with 1 μM VE-821 or DMSO for 1 hour before irradiation, and were irradiated with 3 Gy of carbon ions or 6 Gy of X-rays. They were harvested at 12 and 24 hours after irradiation and their cell cycle distributions were analyzed by flow cytometry. Error bars represent SEM of at least three independent experiments. (PDF 621 kb)

## Abbreviations

ATR: ataxia telangiectasia mutated and Rad3-related
LET: linear energy transfer
RBE: relative biological effectiveness
SER: sensitizing enhancement ratio
