# Supplementary figures and images for: VE-821, an ATR inhibitor, causes radiosensitization in human tumor cells irradiated with high LET radiation
Source: Radiat Oncol. 2015 Aug 19;10:175. doi: 10.1186/s13014-015-0464-y (PMC4554350; doi:10.1186/s13014-015-0464-y)

**Figure S1**

**1BR-  
hTERT**

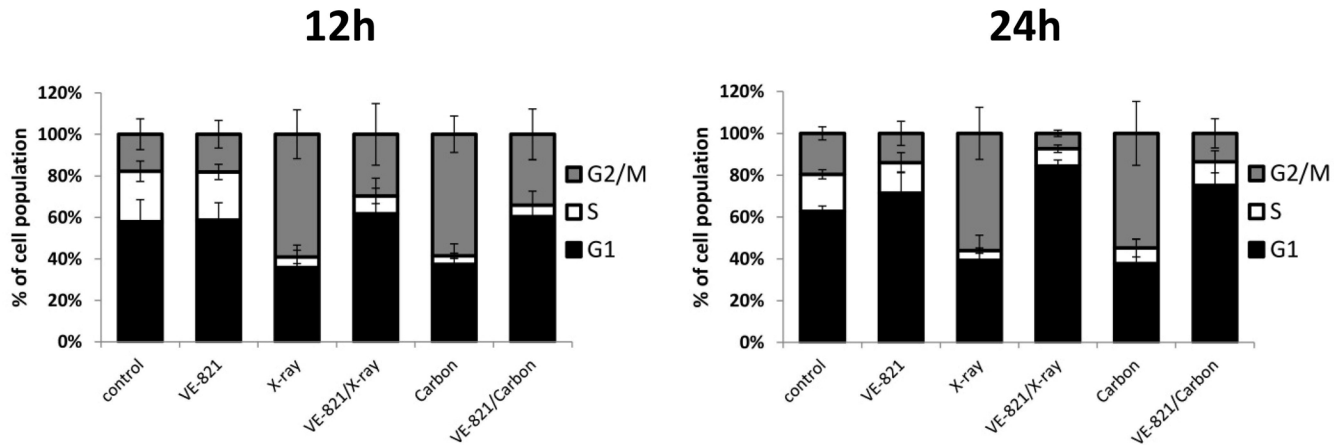

Supplement: Additional file 1: Figure S1. — VE-821 abrogated carbon ion-induced G2/M cell cycle arrest in 1BR-hTERT cells. 1BR-hTERT cells were pre-treated with 1 μM VE-821 or DMSO for 1 hour before irradiation, and were irradiated with 3 Gy of carbon ions or 6 Gy of X-rays. They were harvested at 12 and 24 hours after irradiation and their cell cycle distributions were analyzed by flow cytometry. Error bars represent SEM of at least three independent experiments. (PDF 621 kb) [file 13014_2015_464_MOESM1_ESM.pdf]
